# Y-Chromosome Evidence for Common Ancestry of Three Chinese Populations with a High Risk of Esophageal Cancer

**DOI:** 10.1371/journal.pone.0011118

**Published:** 2010-06-15

**Authors:** Haihua Huang, Min Su, Xiaoyun Li, Hui Li, Dongping Tian, Yuxia Gao, Yubai Guo

**Affiliations:** 1 The Key Immunopathology Laboratory of Guangdong Province, Department of Pathology, Shantou University Medical College, Shantou, Guangdong, China; 2 Second Affiliated Hospital of Shantou University Medical College, Shantou, Guangdong, China; 3 State Key Laboratory of Genetic Engineering and Center for Anthropological Studies, School of Life Sciences, Fudan University, Shanghai, China; 4 Department of Genetics, School of Medicine, Yale University, New Haven, Connecticut, United States of America; Ohio State University Medical Center, United States of America

## Abstract

High rates of esophageal cancer (EC) are found in people of the Henan Taihang Mountain, Fujian Minnan, and Chaoshan regions of China. Historical records describe great waves of populations migrating from north-central China (the Henan and Shanxi Hans) through coastal Fujian Province to the Chaoshan plain. Although these regions are geographically distant, we hypothesized that EC high-risk populations in these three areas could share a common ancestry. Accordingly, we used 16 East Asian-specific Y-chromosome biallelic markers (single nucleotide polymorphisms; Y-SNPs) and six Y-chromosome short tandem repeat (Y-STR) loci to infer the origin of the EC high-risk Chaoshan population (CSP) and the genetic relationship between the CSP and the EC high-risk Henan Taihang Mountain population (HTMP) and Fujian population (FJP). The predominant haplogroups in these three populations are O3*, O3e*, and O3e1, with no significant difference between the populations in the frequency of these genotypes. Frequency distribution and principal component analysis revealed that the CSP is closely related to the HTMP and FJP, even though the former is geographically nearer to other populations (Guangfu and Hakka clans). The FJP is between the CSP and HTMP in the principal component plot. The CSP, FJP and HTMP are more closely related to Chinese Hans than to minorities, except Manchu Chinese, and are descendants of Sino-Tibetans, not Baiyues. Correlation analysis, hierarchical clustering analysis, and phylogenetic analysis (neighbor-joining tree) all support close genetic relatedness among the CSP, FJP and HTMP. The network for haplogroup O3 (including O3*, O3e* and O3e1) showed that the HTMP have highest STR haplotype diversity, suggesting that the HTMP may be a progenitor population for the CSP and FJP. These findings support the potentially important role of shared ancestry in understanding more about the genetic susceptibility in EC etiology in high-risk populations and have implications for determining the molecular basis of this disease.

## Introduction

The non-recombining portion of the Y chromosome (NRY) has unique characteristics, including paternal inheritance, absence of recombination at meiosis, and a relatively low probability of recurrent mutations, thus endowing it with population- and area-specific polymorphisms. Thus, NRY is particularly useful for the study of human evolution and population genetics. Two types of polymorphisms exist on the NRY: single nucleotide polymorphisms (SNPs) and short tandem repeat (STR) loci, each with different mutation rates and mechanisms. Accordingly, combined analysis using these 2 types of polymorphic markers increases the power of the NRY for use in tracing human evolution as well as migration through different geographic locales and time scales, and therefore could also be effective in depicting the paternal structures of populations.

Indeed, in 1999, Su et al. [1] ascertained 17 Y-chromosome haplogroups based on 19 East Asian-specific biallelic markers that reveal the paternal structures of populations in East Asia. By investigating 63 population samples from Asia, Africa, America, Europe, and Oceania, the authors found that southeast populations in East Asia are more genetically diverse than are northern ones and suggested that East Asians originated from the south. On the basis of Y-SNP and Y-STR variance, they concluded that the initial settlement of modern humans in East Asia occurred about 18,000–60,000 years ago [1].

**Figure 1:**
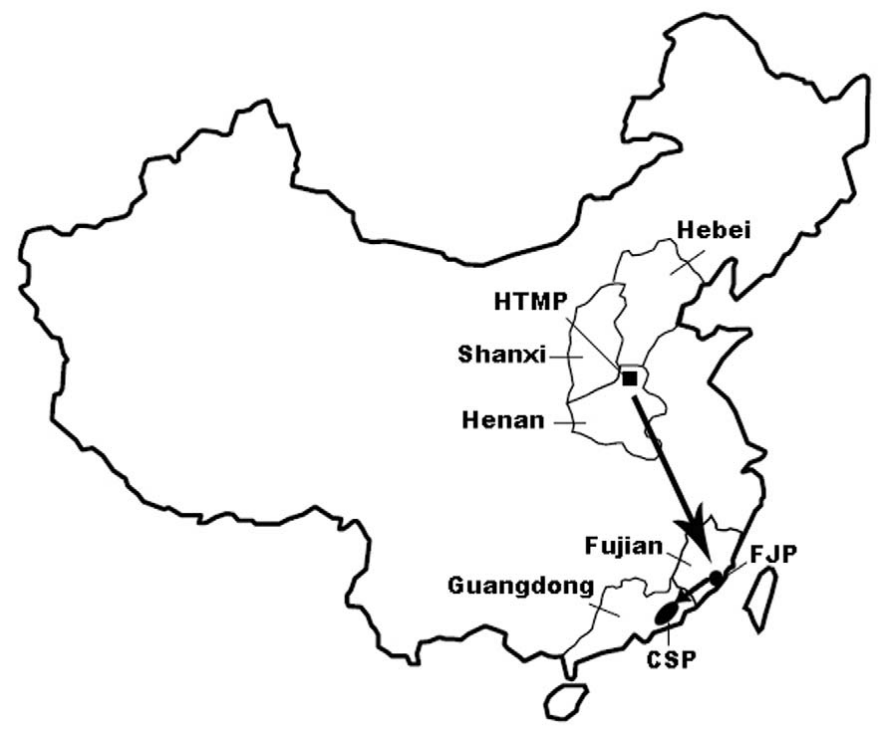
Geographic distribution of the three studied EC high-risk populations. Arrows show the north-to-south migrations of Han inhabitants from north-central China according to historical records. CSP, Chaoshan EC high-risk population; FJP, Fujian EC high-risk population; HTMP, Henan Taihang Mountain EC high-risk population.

Esophageal cancer (EC) is one of the most common fatal cancers worldwide [2] and has high incidences in some geographical regions. In China, most EC patients live in the so-called “EC belt,”, which stretches from central China westward through Central Asia to northern Iran [3]–[4]. The best-known region for high EC risk is the north-central Henan Taihang Mountain area (the HTM population [HTMP]), situated among the Henan, Hebei, and Shanxi Provinces (Fig. 1). Less well-known regions are the southeastern littoral Chaoshan Plain in Guangdong Province (the CS population [CSP]) and the Minnan area of Fujian Province (the FJ population [FJP]). Although the 2 latter provinces are relatively geographically isolated from the interior of China, they are still considered to reside in the EC belt and evidence exists for a high EC risk in these areas [5]–[6]. For example, in a relatively isolated district within the CSP—Nanao Island—annual average crude incidence of EC was 103.98/100,000 people from 1995 to 2004; the age-standardized incidence rates for EC were 72∼150/100,000 males and 26∼64/100,000 females, with a significantly increased incidence for males, but not females between 1995 and 2004 [5]. For the FJP, 18 counties reported a mortality rate for EC greater than 30/100,000 people [7], which is at least twice the national average [8]. The CSP, FJP and HTMP is belong to Northeast-Asian groups.

The geographies of south-littoral (Chaoshan and Fujian areas) and north-central China (Henan and Shanxi) are distinct, but populations within these regions share a high risk of EC [9]. According to historical records [10]–[11], Han inhabitants of north-central China (Henan and Shanxi Hans) continuously migrated into the Chaoshan area through Fujian Province to escape war and famine, and gradually became the predominant inhabitants of Chaoshan. Moreover, familial aggregation of EC has been observed in north-central China and the Chaoshan area [12]–[14]. These findings support the potentially important role of shared ancestry contributing to the genetic susceptibility for EC in high-risk populations in China.

Therefore, we hypothesized that these three EC high-risk populations may be genetically related. To test our hypothesis of common ancestry, we analyzed 16 East Asian-specific biallelic markers [1], [15] (SNPs) and seven Y-STR loci were analyzed to examine the NRY structure in the EC high-risk CSP, FJP and HTMP. Indeed, haplogroup frequencies and principal component, correlation, hierarchical clustering, phylogenetic (neighbor-joining tree) and network analyses all support the close genetic relatedness of the CSP, FJP and HTMP. Ascertaining the genetic background of EC patients can help clarify the molecular genetic mechanisms of esophageal carcinogenesis, improve the evaluation of risk factors in individuals from high-risk populations, and promote the implementation of effective screening and preventive measures.

## Results

### Distribution of NRY haplogroups in the three EC high-risk populations in China

The haplogroup frequencies of the three EC high-risk populations were based on Y-SNP typing. As shown in Table 1, 10 Y- chromosome haplogroups were identifed. The Y-chromosome haplogroups of the three studied populations mainly cluster around O3*, O3e*, and O3e1, which are the characteristic haplogroups for Northern East Asians; the overall frequencies were 65.16%, 66.21% and 60.42% for the CSP, FJP and HTMP, respectively, and did not significantly differ among the populations (2-sided *X* ^2^ = 4.213, *p* = 0.122). These results provide evidence for genetic affinity between these three EC high-risk populations. O*, O1*, O2a*, and O2a1, the four common haplogroups in the southern East Asians, were more frequent in the CSP and HTMP than in the FJP (2-sided *X* ^2^ = 8.355, *p* = 0.015). The C*, D1, and F* haplogroups were more common in the northern than southern group, and their combined frequencies were significantly lower in the CSP than in the other two (2-sided *X* ^2^ = 11.327, *p* = 0.003). These results support the hypothesis of gene flow between the HTMP, FJP, and CSP.

**Table 1:** Y-chromosome single nucleotide polymorphism (Y-SNP) haplogroup frequencies of the 3 EC high-risk populations (%).

Haplogroup	Chaoshan EC high-risk population(n = 89)	Fujian EC high-risk population(n = 74)	Henan Taihang Mountain EC high-risk population(n = 48)
O^*^	13.48	5.41	2.08
O1^*^	10.11	1.35	4.17
O2a^*^	2.25	2.70	10.42
**O2a1**	1.12	0.00	0.00
O3^*^	29.21	24.32	14.58
O3e^*^	10.11	9.46	29.17
**O3e1**	25.84	32.43	16.67
C^*^	3.37	2.70	10.42
**D1**	0.00	1.35	0.00
F^*^	4.49	20.27	12.5

### PC analysis reveals close affinity among the three EC high-risk populations

**Figure 2:**
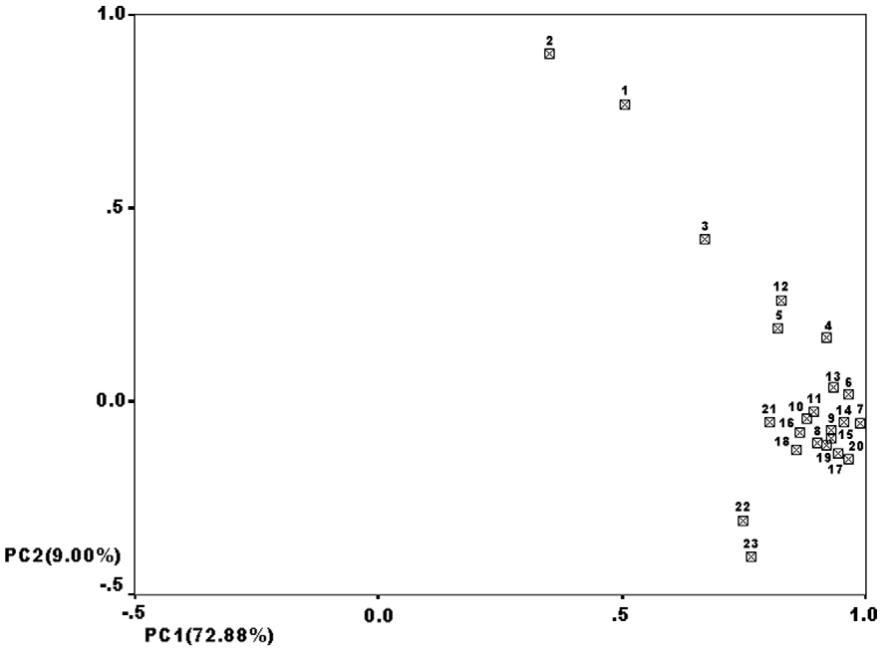
The two-dimensional maps of Y-SNP frequencies. In the principal-component plot, the smaller the distance between populations, the closer their relationship. In general 23 populations are divided into 4 clusters: the three EC high-risk populations, two Northern Han populations (labeled 5 and 6) and Hakka population, two Southern Han populations (labeled 22 and 23) and the rest of the Northern Han and Southern Han cluster, respectively. 1, Chaoshan EC high-risk population; 2, Fujian EC high-risk population; 3, Henan Taihang Mountain EC high-risk population; 4, Hebei Han; 5, Liaoning Han; 6, Xinjiang Han; 7, Shangdong Han; 8, Gansu Han; 9, Shanxi Han; 10, Neimeng Han; 11, Henan Han; 12, Hakka Han; 13, Hunan Han; 14, Hubei Han; 15, Guangzhou Han; 16, Zhejiang Han; 17, Jiangxi Han; 18, Shanghai Han; 19, Anhui Han; 20, Jiangsu Han; 21, Yunnan Han; 22, Guangxi Han; 23, Sichuan Han. 4–11 refer to Northern Han and 12–23 to Southern Han.

**Figure 3:**
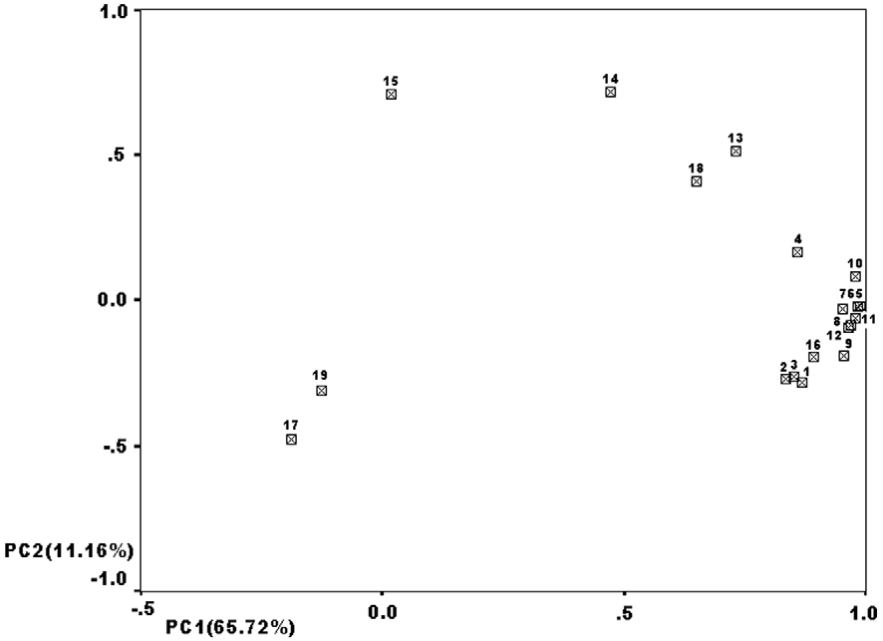
The two-dimensional graphs of Y-STR frequencies. This map accounts for 76.88% of the original genetic variation. The three EC high-risk populations and Manchu form 2 clusters; the three Northern Han (labeled 10–12) and five Southern Han populations (labeled 5–9) form another group. The remaining populations are scattered. 1, Chaoshan EC high-risk population; 2, Fujian EC high-risk population; 3, Henan Taihang Mountain EC high-risk population; 4, Fujian Han; 5, Anhui Han; 6, Yunnan Han; 7, Henan Han; 8, Zhejiang Han; 9, Guangzhou Han; 10, Dongbei Han; 11, Beijing Han; 12, Tianjing Han; 13, Tibetan; 14, Uygur; 15, Krigiz; 16, Manchu; 17, Shui; 18, Naxi; 19, Zhuang. 4–9 refer to Southern Hans, 10–12 to Northern Hans, 13–17 to Northern minority nationalities, 18–19 to Southern minority nationalities.

PC plots of Y-SNP and Y-STR frequencies are shown in Figs. 2 and 3, respectively, and are based on genotyping results of the three EC high-risk populations and additional data from other Chinese populations. Figs. 2 and 3 are the two-dimensional graphs for component 1(PC1) and component 2 (PC2). The cumulative contribution of PC1 and PC2 accounted for 79.88% and 76.88%, respectively, of the total variations from Y-SNP and Y-STR, respectively. As shown in Fig. 2, the three EC high-risk populations mainly form a cluster. Two Northern Hans populations (labeled 4 and 5) and the Hakka (labeled 13) population, two Southern Hans (labeled 22 and 23) cluster together, respectively. The rest of the Northern Hans and Southern Hans form another group. The EC high-risk CSP is isolated from both the Guangzhou Han (labeled 15) and Hakka populations. The three EC high-risk populations and Manchu form a distinct cluster (Fig. 3). They are more closely related to Chinese Hans than to the other six minorities. Six minority nationalities and one Southern Han (labeled 4) are scattered. The rest of the Northern and Southern Hans form another group.

### Correlation analysis reveals positive associations between the three EC high-risk populations and Chinese Hans

To further elucidate the relationship between populations, we performed correlation analysis based on Y-SNP haplogroup and Y-STR haplotype frequencies (Tables 2 and 3, respectively). Significant positive correlation existed among the three EC high-risk populations. The correlation coefficient between the EC high-risk CSP and FJP was higher than that between the CSP and the EC high-risk HTMP. Moreover, Table 2 shows a positively correlated between the HTMP and most of the Chinese Hans, a positive correlation between the FJP and only the Liaoning Han, and a positive correlation between the CSP and the Hakka, Hebei, and Hunan Hans (Table 2). Because of poor communication and transportation in past, Chaoshan and Fujian areas may have become a relatively closed society, with less gene flow with other populations, and because the EC high-risk HTMP is located in central China, this population may have had more opportunity to communicate with other Chinese Hans. Table 3 shows a positive correlation between the three high-risk populations and the Chinese Hans. Unexpectedly, the data show a positive correlation between the three studied populations and the Manchu, which is a northern minority population. No significant correlation was found between other populations and the three EC high-risk populations. The discrepancy between data in Tables 2 and 3 may be related to the compared populations and the different characteristics of STRs and SNPs. SNPs are characterized by low mutation rate and low probabilities of back and parallel mutation, making SNP analysis suitable for tracing early demographic events in human history. The mutation rate of STRs is much higher than that of SNPs and, therefore, the former are suitable for investigating details of demographic events occurring on a more recent time-scale.

**Table 2:** Correlation analysis of Y-SNP haplogroup frequencies of the 3 EC high-risk populations and 20 Chinese Han populations.

	Chaoshan EC high-risk population	Fujian EC high-risk population	Taihang Mountain EC high-risk population
**Fujian EC high-risk population**	0.85**		
**Taihang Mountain EC high-risk population**	0.60*	0.67**	
**Hakka**	0.61**	0.44	0.42
**Hebei Han**	0.57*	0.41	0.57*
**Shangdong Han**	0.45	0.29	0.61**
**Henan Han**	0.45	0.28	0.35
**Anhui Han**	0.46	0.21	0.45
**Zhejiang Han**	0.48	0.21	0.61**
**Jiangsu Han**	0.43	0.21	0.52*
**Shanghai Han**	0.46	0.18	0.36
**Hebei Han**	0.48	0.26	0.65**
**Sichuan Han**	0.06	−0.02	0.52*
**Jiangxi Han**	0.35	0.26	0.60*
**Hunan Han**	0.53*	0.31	0.61**
**Yunnan Han**	0.30	0.23	0.82**
**Gansu Han**	0.30	0.25	0.55*
**Liaoning Han**	0.41	0.50*	0.56*
**Neimeng Han**	0.33	0.27	0.68**
**Shanxi Han**	0.33	0.29	0.63**
**Xinjiang Han**	0.45	0.34	0.62**
**Guangdong Han**	0.40	0.22	0.68**
**Guangxi Han**	0.24	0.02	0.40
**Yunnan Han**	0.47	0.35	0.61**

**P<0.01 (2-tailed).

*P<0.05 (2-tailed).

**Table 3:** Correlation analyses of Y- short tandem repeat (Y-STR) haplotype frequencies of the 3 EC high-risk populations, 9 Chinese Han populations, and 7 Chinese minority nationalities.

	Chaoshan EC high-risk population	Fujian EC high-risk Population	Taihang Mountain EC high-risk population
**Fujian EC high-risk population**	0.94**		
**Taihang Mountain EC high-risk population**	0.93**	0.87**	
**Zhejiang Han**	0.54**	0.49**	0.59**
**Dongbei Han**	0.84**	0.77**	0.82**
**Tianjing Han**	0.72**	0.57**	0.72**
**Henan Han**	0.81**	0.75**	0.81**
**Tibetan**	0.44*	0.33	0.45*
**Fujian Han**	0.53**	0.45*	0.63**
**Anhui Han**	0.73**	0.63**	0.75**
**Beijing Han**	0.77**	0.67**	0.75**
**Guangzhou Han**	0.63**	0.52**	0.62**
**Yunnan Han**	0.86**	0.80**	0.85**
**Naxi**	0.25	0.05	0.15
**Shui**	0.11	−0.03	−0.03
**Manchu**	0.64**	0.58**	0.73**
**Uygur**	0.11	0.02	0.28
**Kirgiz**	−0.23	−0.32	−0.13
**Zhuang**	0.02	−0.01	0.16

**P<0.01 level (2-tailed).

*P<0.05 level (2-tailed).

### The three EC high-risk populations cluster in the Sino-Tibetan language family

To delineate the genetic relationship among the three EC high-risk populations and populations based on other language families, we performed a further principal component analysis using data, provided by the State Key Laboratory of Genetic Engineering and Center for Anthropological Studies (School of Life Sciences, Fudan University), which include Y-SNP data for 64 Chinese populations belonging to the 5 language families: Sino-Tibetan, Hmong-Mien, Daic (Baiyue), Austronesian, and Austroasiatic.

**Figure 4:**
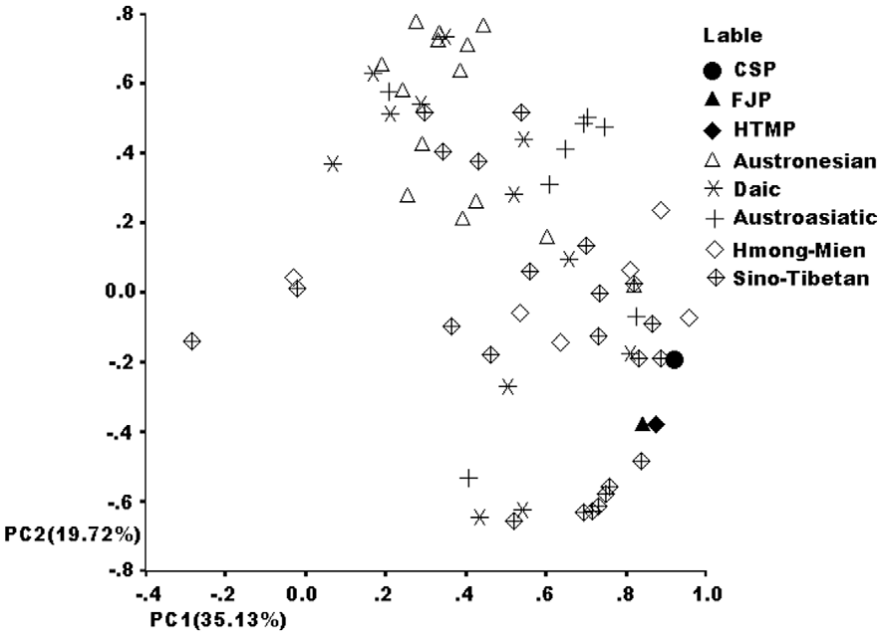
Principal component dot plot of Y-chromosome haplogroups. Chaoshan, Henan Taihang Mountain and Fujian EC high-risk populations are clustered together and located among Sino-Tibetan populations, which suggests a close genetic relationship among them.

The Y-chromosome haplogroup profiles identified in these populations were treated as input vectors for PC analysis. The cumulative contribution of PC1 and PC2 accounted for 54.85% of the total variance. A PC dot plot was drawn with values for PC1 and PC2 as the X and Y axes, respectively. As shown in Fig. 4, PC1 values are associated with a tighter clustering of populations belonging to the Sino-Tibetan language family; high PC2 values are associated with a more scattered distribution of Sino-Tibetan populations. The three EC high-risk populations are clustered in the rightmost part of the PC plot, among the most intense distribution of Sino-Tibetans.

Correlation analysis was carried out to seek the origin of each PC (Table 4). PC1 showed a significant negative correlation with PC2 (r = −0.44, *P*<0.001). Analysis of PC1 showed that the number of negatively correlated haplogroups, despite their weak correlations, was larger than that of the positive groups. O3e* is the only haplogroup with a significantly positive correlation with PC1 (r = 0.61, *P*<0.05). For PC2, the number of positively and negatively correlated haplogroups was similar. O2a* and O1 represent the southern aboriginal haplogroups in the East Asian population and O3e* is probably a northern haplogroup. As shown in Table 4, the distribution of O3e*, O2a*, and O1 is opposite with PC1 and PC2. O3e* was positively correlated with PC1, but O2a* and O1 were negatively correlated. O2a* and O1 were positively correlated with PC2, but O3e* was negatively correlated. This finding implies that these three haplogroups are the main components of PC1 and PC2. Of note, analysis of only haplogroup O3e* was consistent with the distribution of Sino-Tibetan populations shown in Fig. 4. Furthermore, the three EC high-risk populations are almost at the peak value of PC1 and cluster with some Sino-Tibetan populations, suggesting that the EC high-risk populations are all typical Sino-Tibetan populations.

**Table 4:** Correlation analysis of principal components and Y-SNP frequencies among populations.

		C*	D*	D1	F*	K*	O3*	O3d*	O3e*	O1*	O1b	O2a*	O2a1	Q1	P*	R*	PC1
D*	*r*	0.09															
	*P*	0.23															
**D1**	*r*	−0.05	0.18														
	*P*	0.33	0.07														
F*	*r*	−0.10	−0.04	−0.05													
	*P*	0.21	0.38	0.35													
K*	*r*	0.02	−0.15	−0.08	−0.02												
	*P*	0.42	0.11	0.25	0.44												
O3*	*r*	−0.10	−0.19	−0.10	−0.13	−0.09											
	*P*	0.21	0.07	0.20	0.14	0.24											
O3d*	*r*	0.25	−0.08	−0.09	−0.18	−0.07	−0.09										
	*P*	0.02	0.25	0.23	0.07	0.30	0.22										
O3e*	*r*	−0.16	0.10	−0.09	0.03	−0.35	−0.27	−0.03									
	*P*	0.10	0.20	0.25	0.42	0.00	0.02	0.41									
O1*	*r*	−0.07	−0.16	−0.15	−0.08	0.08	−0.09	−0.05	−0.31								
	*P*	0.27	0.10	0.12	0.27	0.26	0.24	0.33	0.01								
**O1b**	*r*	−0.12	−0.10	0.02	0.16	−0.05	−0.05	−0.10	−0.19	0.29							
	*P*	0.17	0.22	0.45	0.10	0.35	0.33	0.22	0.07	0.01							
O2a*	*r*	−0.14	−0.13	0.09	−0.02	−0.06	−0.22	−0.11	−0.41	0.20	0.06						
	*P*	0.13	0.15	0.24	0.43	0.32	0.04	0.18	0.00	0.05	0.31						
**O2a1**	*r*	−0.08	0.37	−0.09	−0.11	−0.22	−0.16	−0.08	−0.09	−0.01	−0.04	0.03					
	*P*	0.25	0.00	0.23	0.18	0.04	0.10	0.26	0.23	0.48	0.39	0.41					
**Q1**	*r*	−0.10	−0.04	−0.05	−0.12	−0.14	−0.04	−0.06	−0.15	−0.10	−0.07	−0.15	0.45				
	*P*	0.22	0.36	0.34	0.17	0.12	0.36	0.33	0.11	0.21	0.30	0.12	0.00				
P*	*r*	−0.05	0.03	−0.09	0.04	−0.05	−0.01	−0.10	−0.13	−0.07	0.04	−0.11	−0.03	0.28			
	*P*	0.33	0.42	0.23	0.37	0.35	0.46	0.21	0.15	0.29	0.38	0.19	0.39	0.01			
R*	*r*	−0.02	0.08	0.10	−0.04	0.11	−0.04	−0.08	−0.12	−0.01	−0.08	−0.03	−0.06	−0.05	0.31		
	*P*	0.45	0.25	0.21	0.38	0.19	0.38	0.25	0.18	0.45	0.27	0.41	0.31	0.36	0.01		
**PC1**	*r*	−0.15	−0.06	−0.20	0.08	−0.08	0.15	−0.17	0.61	−0.13	−0.21	−0.23	−0.24	−0.45	−0.28	−0.02	
	*P*	0.11	0.33	0.05	0.26	0.27	0.11	0.09	0.00	0.16	0.05	0.03	0.03	0.00	0.01	0.43	
**PC2**	*r*	−0.03	−0.32	0.00	−0.06	0.43	0.14	−0.10	−0.84	0.44	0.23	0.66	−0.08	−0.07	−0.02	0.11	−0.44
	*P*	0.39	0.00	0.49	0.30	0.00	0.13	0.21	0.00	0.00	0.03	0.00	0.25	0.28	0.45	0.19	0.00

*r*: correlation coefficient; *P*: probability value. PC1: principal component 1. PC2: principal component 2. O3e*, a predominant characteristic haplogroup of Hans, was positively associated with PC1 but negatively associated with PC2. In contrast, O2a*, a typical southern aboriginal haplogroup in East Asian populations, was negatively associated with PC1 but positively associated with PC2.

### Hierarchical cluster analysis reveals isolation of the EC high-risk cluster from other populations

**Figure 5:**
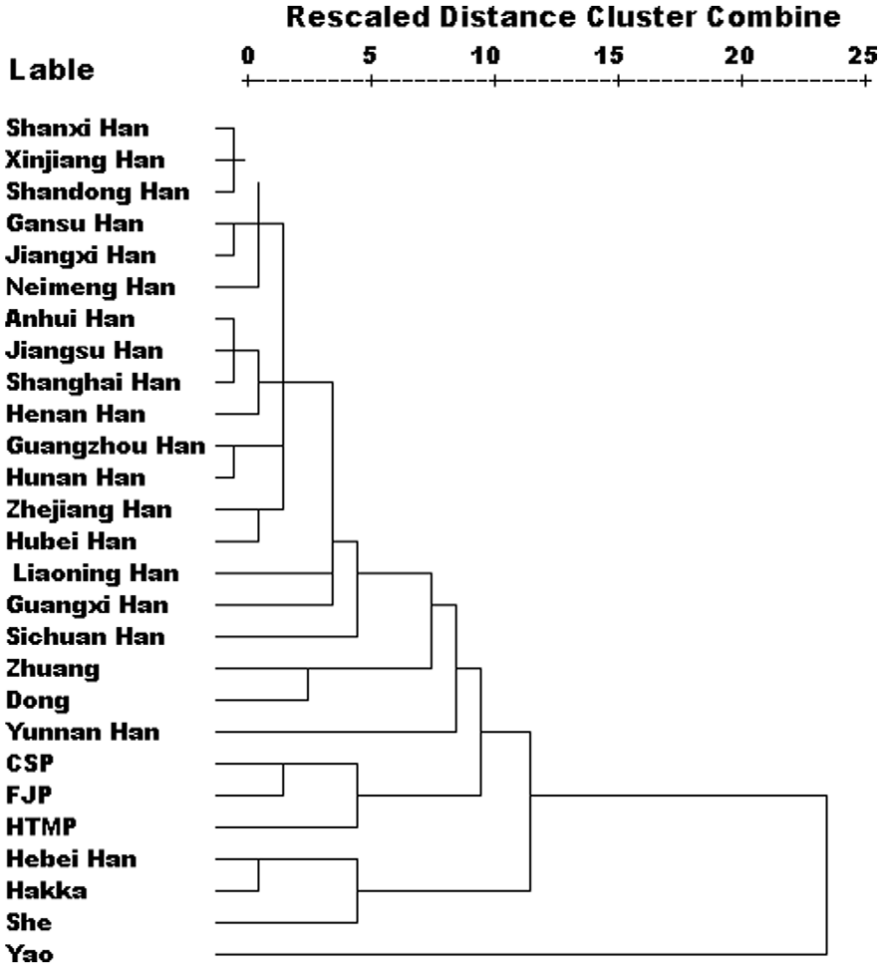
Dendrogram based on Y-SNP data. Shows the affinity between the three EC high-risk populations. Most Chinese Hans are grouped into a large cluster in the upper part of the dendrogram.

To further elucidate the affinity among the three EC high-risk populations and other Chinese populations, hierarchical cluster analysis was carried out with average linkage (between groups) based on Y-SNP data. To illustrate the cluster of the three high-risk populations and its relationship with other Hans, Baiyue, and Hmong-Mien groups, we included 20 Chinese Hans [16]−[18], data for Liaoning, Guangzhou and Guangxi Hans was provided by the State Key Laboratory of Genetic Engineering and Center for Anthropological Studies (School of Life Sciences, Fudan University), two Hmong-Mien (She [19] and Yao[1]) and two Baiyue populations (Zhuang and Dong) [1] (Fig. 5). The information of the data for comparison is shown in Table 5. Hierarchical clustering analysis elucidates the genetic distance among populations. In Fig. 5, the EC high-risk CSP is genetically close to the EC high-risk FJP and HTMP. The genetic distance between FJP and CSP is shortest. Moreover, the three are isolated from other populations, which implies a particular migration event in ancient times.

**Table 5:** Y-chromosome haplogroup frequency distribution in the 3 EC high-risk populations, 20 Chinese Hans and 4 minority nationalities.

populations	Label in fig. 2 and 3	size	C	D/E	D1	F	K*	O3*	O3d	O3e*	O*	O1*	O1b	O2a*	O2a1	Q1	P*	O3e1
**Northern Hans**																		
**HTMP** *	3	48	10.42	0.00	0.00	12.50	0.00	14.58	0.00	29.17	2.08	4.17	0.00	10.42	0.00	0.00	0.00	16.67
**Hebei^a^**	4	6	0.00	0.00	0.00	0.00	16.70	50.00	0.00	33.30	0.00	0.00	0.00	0.00	0.00	0.00	0.00	0.00
**Liaoning** #	5	48	2.08	2.08	0.00	22.92	16.67	27.08	0.00	18.75	0.00	4.17	0.00	2.08	0.00	4.17	0.00	0.00
**Xinjiang^c^**	6	51	3.92	1.96	0.00	5.88	17.65	29.41	0.00	29.41	0.00	3.92	0.00	0.00	0.00	3.92	3.92	0.00
**Shangdong^a^**	7	32	9.40	0.00	0.00	3.10	18.80	28.10	0.00	28.10	0.00	9.40	0.00	0.00	0.00	3.10	0.00	0.00
**Gansu^c^**	8	60	11.67	8.33	0.00	10.00	16.67	18.33	0.00	18.33	0.00	8.33	0.00	1.67	0.00	5.00	1.67	0.00
**Shanxi^c^**	9	90	2.22	3.33	0.00	7.78	22.22	23.33	0.00	33.33	0.00	2.22	0.00	1.11	0.00	2.22	2.22	0.00
**Neimeng^c^**	10	60	20.00	5.00	0.00	6.67	13.33	21.67	0.00	26.67	0.00	1.67	0.00	1.67	0.00	3.33	0.00	0.00
**Henan^a^**	11	28	7.10	0.00	0.00	3.60	25.00	32.10	0.00	14.30	0.00	10.70	0.00	0.00	0.00	7.10	0.00	0.00
**Southern Hans**																		
**CSP** *	1	89	3.37	0.00	0.00	4.49	0.00	29.21	0.00	10.11	13.48	10.11	0.00	2.25	1.12	0.00	0.00	25.84
**FJP** *	2	74	2.70	0.00	1.35	20.27	0.00	24.32	0.00	9.46	5.41	1.35	0.00	2.70	0.00	0.00	0.00	32.43
**Hakka^b^**	12	148	2.70	0.68	0.00	2.03	14.19	54.05	4.05	16.22	0.00	2.03	0.68	2.70	0.00	0.68	0.00	0.00
**Hunan^a^**	13	15	0.00	0.00	0.00	0.00	13.33	33.33	0.00	26.67	0.00	13.33	0.00	13.33	0.00	0.00	0.00	0.00
**Hubei^a^**	14	18	5.60	0.00	0.00	0.00	11.11	27.78	5.56	33.33	0.00	16.67	0.00	0.00	0.00	0.00	0.00	0.00
**Guangdong** #	15	64	4.69	0.00	1.56	0.00	12.50	23.44	0.00	29.69	0.00	7.81	0.00	10.94	7.81	1.56	0.00	0.00
**Zhejiang^a^**	16	50	12.00	0.00	0.00	0.00	6.00	24.00	0.00	26.00	0.00	26.00	0.00	6.00	0.00	0.00	0.00	0.00
**Jiangxi^a^**	17	21	4.76	4.76	0.00	9.52	19.05	19.05	0.00	23.81	0.00	14.29	0.00	4.76	0.00	0.00	0.00	0.00
**Shanghai^a^**	18	30	6.70	0.00	0.00	0.00	16.70	26.70	3.30	16.70	0.00	26.70	0.00	0.00	0.00	3.30	0.00	0.00
**Anhui^a^**	19	22	13.64	0.00	0.00	0.00	18.18	27.27	0.00	18.18	0.00	18.18	0.00	0.00	0.00	4.55	0.00	0.00
**Jiangsu^a^**	20	55	7.30	1.80	0.00	1.80	18.20	23.60	3.60	21.80	0.00	16.40	0.00	3.60	0.00	1.80	0.00	0.00
**Yunnan^a^**	21	27	11.10	0.00	0.00	3.70	3.70	18.50	0.00	55.60	0.00	3.70	0.00	3.70	0.00	0.00	0.00	0.00
**Guangxi** #	22	26	7.69	0.00	0.00	0.00	15.38	15.38	0.00	19.23	0.00	15.38	0.00	7.69	19.23	0.00	0.00	0.00
**Sichuan^a^**	23	14	7.10	0.00	0.00	0.00	28.60	7.10	7.10	35.70	0.00	7.10	0.00	7.10	0.00	0.00	0.00	0.00
**Zhuang^d^**		28	3.60	0.00	3.60	7.10	3.60	3.60	0.00	25.00	17.90	0.00	25.00	10.70	0.00	0.00	0.00	0.00
**Dong^d^**		10	20.0	0.00	0.00	0.00	0.00	10.0	0.00	20.00	20.00	10.0	20.00	0.00	0.00	0.00	0.00	0.00
**Yao^d^**		10	20.0	30.00	0.00	0.00	0.00	10.0	0.00	0.00	0.00	0.00	40.00	0.00	0.00	0.00	0.00	0.00
**She-Fujian^e^**		26	0.00	0.00	0.00	0.00	11.50	65.50	0.00	3.80	11.50	0.00	7.70	0.00	0.00	0.00	0.00	0.00

# Data provided by the State Key Laboratory of Genetic Engineering and Center for Anthropological Studies, School of Life Sciences, Fudan University.

*Data of the three study EC high-risk population; a from reference 17; b from reference 18; c from reference 16; d from reference 1; e from reference 19.

### Genetic distance analysis and construction of a phylogenetic tree

**Figure 6:**
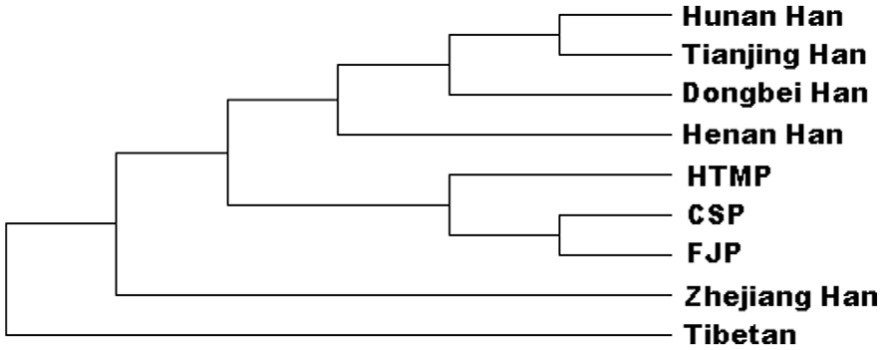
A neighbor-joining tree of Chinese populations based on Y-STR data. This unrooted tree was constructed by use of genetic distances between populations. Shows that the EC high-risk Chaoshan is extremely close to the EC high-risk FJP and that the 2 are clustered with the EC high-risk HTMP. Hunan, Tianjing, Dongbei, and Henan Hans are grouped in the upper part of the tree.

To further investigate the genetic relationships between the three EC high-risk populations, Rst distances between pairs of populations were calculated on the basis of seven Y-STRs by use of Alrequin 3.1 software. Six additional Chinese populations were included in this analysis: Zhejiang [20], Henan [21], Dongbei [22], Tianjing [23], and Hunan Hans [24], and Tibetan [25], all of which belong to the Sino-Tibetan language family, as do the three EC high-risk populations. From the Rst distance matrix, an unrooted neighbor-joining tree was constructed with use of MEGA 2.1 software (Fig. 6). The EC high-risk CSP was closely related to the EC high-risk FJP and HTMP. All three are closer to Chinese Hans than to the Tibetan population. The Hunan, Tianjing, Dongbei, and Henan Hans are grouped together.

### Network analysis of Y-STR haplogroups of the three EC high-risk populations

**Figure 7:**
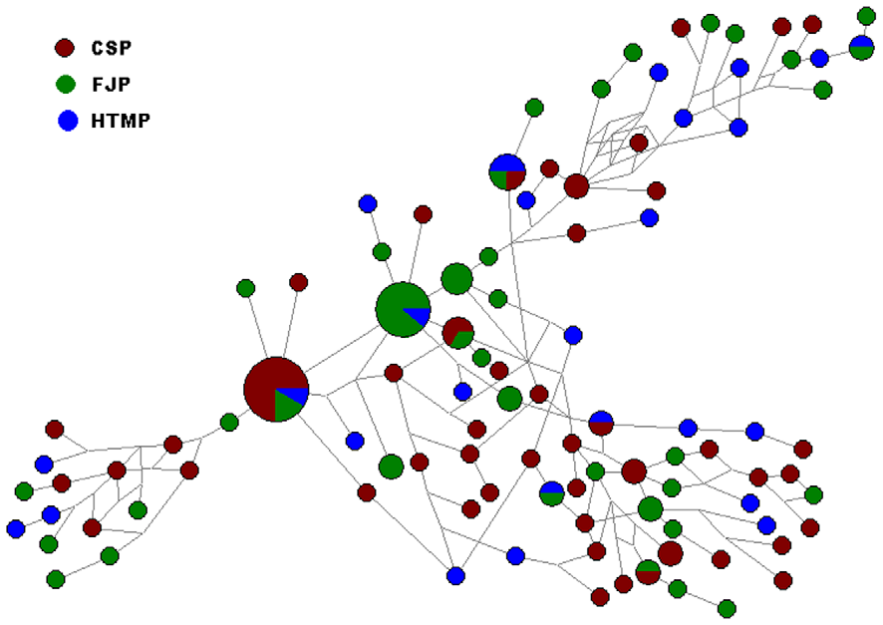
STR network of haplogroups O3*, O3e* and O3e1 of the three EC high-risk populations. In the network, HTMP have more STR haplogroup polymorphisms than CSP and FJP. Six Y-STRs of HTMP are share by CSP and/or FJP. Circles represent lineages, area is proportional to frequency, and color indicates population of origin.

The highest haplogroup frequency shared by the CSP, FJP and HTMP was haplogroup O3. The network for haplogroup O3 was further constructed using Network 4.516 software (www.fluxus-engineering.com) based on all of haplogroup O3 (including O3*, O3e* and O3e1*) individuals for analyzing the relationship among CSP, FJP and HTMP. As shown in Fig. 7, 29 individuals from HTMP have 26 Y-STRs, the highest Y- STRs frequency of all, six of which are shared by CSP and/or FJP. 58 individuals from CSP have 47 Y- STRs, 49 individuals from FJP have 37 Y- STRs. Y- STR frequency of the CSP is slightly higher than that of the FJP, which may be due to the geographical proximity of these two areas, and more frequent gene flow between them.

## Discussion

Our hypotheses for this study, that EC high-risk populations in the Henan Taihang Mountain, Fujian Minnan, and Chaoshan regions of China could share a common ancestry, was based on historical records of migration across China. In southern China (Guangdong district) the Baiyue populations formed the earliest settlement in modern history. Before 2200 BC, one branch of the Baiyue population—the Minyue—was the main group living in the Chaoshan littoral areas. The north-to-south strategic expansion started by Emperor Qin Shi Huang initiated large southward migrations of central China Hans from 214 BC onward [26]. During the Han Dynasty (206 BC∼220 AD), three waves of large-scale migrations into southern China resulted in a decrease in the native population in this area. As recorded in pedigrees and ancient inscriptions during the Northern Song Dynasty (960∼1127 AD), large numbers of southern Fujian people, especially from Quanzhou and Putian, settled in the Chaoshan area [27]. Gradually, over a period of 2000 years, this became the major population in the Chaoshan region (called the Helao or Fulao peoples), coming largely from Henan and Shanxi via Fujian with well-maintained language and customs from north-central China. Because of geographic isolation and the historical difficulty in traveling, the Helao/Fulao became a relatively isolated population. Currently, most Fujian and Chaoshan populations believe they are descendants of north-central China Hans, which is supported by genealogical records, stone tablets, and archeological discoveries [28]. Our team has collected and analyzed 40 genealogies of different surnames in Chaoshan areas. Nearly all the ancestors of these 40 genealogies come from North-central China. Most of them first settled in Fujian, then migrated to the Chaoshan area (data unpublished). This result also confirmed the historical record and our hypothesis.

The incidence and mortality rate for EC is very high in the CSP, FJP, and HTMP areas. We propose that the Chaoshan littoral region is an EC high-risk area because of the genetic background of the CSP and FJP shared with those in north-central China. The ancestors of the EC high-risk CSP may have derived from the EC high-risk HTMP via the FJP. This study provides genetic evidence to support this hypothesis

In general, populations sharing similar patterns of haplogroup distribution are likely to have a relatively close genetic relationship. In this study, the three EC high-risk populations resemble one another in distribution of haplogroups O3*, O3e* and O3e1 (Table 1), which suggests they are relatively closely related. PC and correlation analyses were also carried out to further verify the genetic relatedness among the three EC high-risk populations and other groups. As shown in the PC analysis, the paternal structure for the EC high-risk CSP differs from that for the Guangzhou and Hakka Hans, although they are in geographic proximity and all consider themselves descendants of north-central China Hans. In contrast, the EC high-risk CSP closely clustered with the EC high-risk FJP and HTMP, although the HTMP and CSP are geographically disjunct. In addition, correlation analysis based on the Y-SNP haplogroup and Y-STR haplotype frequencies revealed a positive correlation among the three EC high-risk populations, which further supports their close genetic affinity.

As historically recorded 2 millennia ago, southern China was originally inhabited by the southern natives, including those speaking Daic (Baiyue), Austro-Asiatic, and Hmong-Mien languages [29]−[30]. Hence, we included Daic, Hmong-Mien, Sino-Tibetan, Austronesian, and Austroasiatic populations in the PC analysis for comparison. The results in Fig. 4 and the correlation analysis (Table 4) reveal that the EC high-risk CSP is related to Sino-Tibetans, not Baiyues, which is consistent with the migration history of the CSP. The results of hierarchical clustering analysis further support the close genetic affinity among the three EC high-risk populations. Furthermore, our results also revealed that these three populations are closer to the Yunnan Han and to two Baiyue populations (Zhuang and Dong) than to other Chinese Hans, which suggests gene flow among them. The paternal genetic structure of the three EC high-risk populations is distinct from those of other Chinese Hans. The phylogenetic affinity among the three studied populations was further revealed in a neighbor-joining tree (Fig. 6). The network for haplogroup O3 (including O3*, O3e* and O3e1) showed that the HTMP has a higher STR haplotype diversity than the CSP and FJP, while sharing some STR mutations with the CSP and FJP, suggesting the close relationship among them and further suggesting that the HTMP may be a progenitor population for the CSP and FJP. Taken together, these findings support the hypothesis that the EC high-risk CSP shares common genetic traits with the EC high-risk FJP and HTMP, and that they may share a recent common ancestor. Unexpectedly, we found a positive correlation between the three EC populations and the Manchu, a northern minority population. The reason for this affinity is unknown but suggests gene flow between these groups.

Although we used two types of Y-chromosome polymorphic markers and demonstrated consistent results from multiple analyses, populations from other EC high-risk areas were not included in this study, so we cannot ascertain whether all the EC high-risk populations share a common genetic background. To further explore this aspect, a large-scale study of EC high-risk populations is necessary.

In summary, the patrilineal genetic structure of the EC high-risk CSP, HTMP, and FJP suggests an origin in genetic background of the EC high-risk CSP. The three EC high-risk populations in this study appear to share a similar patrilineal genetic background that may explain, at least in part, the high incidence of EC in these areas in China. The extent to which other factors such as environment and customs may contribute to the high incidence of EC remains to be explored.

## Materials and Methods

### Sample collection and DNA extraction

Blood samples of 211 unrelated healthy males were collected from the the three EC high-risk areas in China during 2002 to 2004; 89 samples were from the Chaoshan area, 48 from the Henan Taihang mountain area and 74 from the Fujian area (Minnan area). All individuals gave their informed consent before being included in the study. The study was approved by the ethical review committees of the Medical College of Shantou University. Genomic DNA was extracted from whole blood by standard phenol/chloroform methods [31]. DNA samples were stored at −20°C after extraction.

### Genotyping of Y chromosome SNPs and STRs

**Figure 8:**
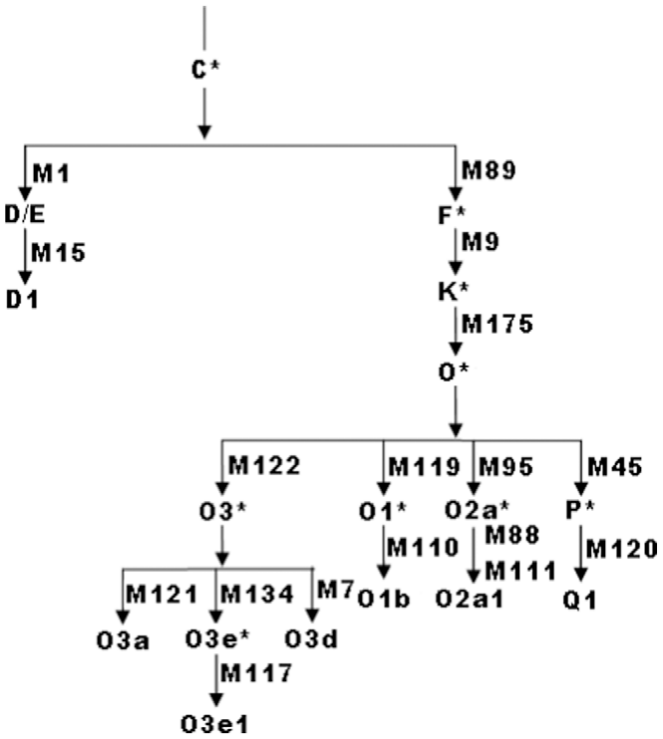
Phylogenetic diagram of 17 haplogroups. This diagram defined by 16 Y chromosome biallelic markers drawn according to the NRY haplogroup tree of East Asia (shown in reference 34). The most recent markers defining the haplogroups are beside the branches.

Three strategies were used to type Y-SNPs and Y-STRs. SNPs without length changes (base substitutions) were genotyped by a PCR-based restriction fragment length polymorphism (PCR-RFLP) method (primer information, restriction enzymes, pattern of polymorphism, and PCR conditions are in Table 6) [17], [32]. SNPs with length variation (e.g., deletion or insertion) were typed by fluorescence PCR (primer information and PCR conditions are in Table 7), and fluorescent-labeled extension products were electrophoresed on a 3100 Genetic Analyzer (ABI company, USA). Y-STRs were also analyzed by this method (Table 7). Analysis of the M1 polymorphism (Alu insertion, also called YAP, see Table 6) was by agarose gel electrophoresis directly after PCR [33]. All primers were synthesized by Sangon Co. (Shanghai, China). Restriction endonucleases were purchased from New England Biolabs, USA. Y-SNP haplogroup assignments were based on the typing results. The phylogenetic diagram of 17 haplogroups defined by 16 Y-SNPs is in Fig. 8 [34].

**Table 6:** Primer sequences and amplification conditions for 10 Y-chromosome SNPs.

Loci	Primer sequence (5′-3′)	Product length	Restriction enzyme	Wild type	Mutant type	Cut
**M1**	F:CAGGGGAAGATAAAGAAATAR:ACTGCTAAAAGGGGATGGAT	150 bp	/	No Alu	Alu insert	/
**PCR conditions**	94°C 3′; 94°C 20″, 51°C* 40″, 72°C 40″, ×35 cycles; 72°C 5′; 4°C for storage.					
**M9**	F:GAAACGGCCTAAGATGGTTGG ATR:AAACTGAATCTTTTT TCCTCATTTTTG	210 bp	BamHI	C	G	C
**M45**	F:ATTGGCAGTGAAAAATTATAGCTAR:TGCCTTTGCTACAACTCTCCTA	162 bp	BfaI	G	A	G
**M89**	F:GAAAGT GGG GCCCACAGAAGGAR:GCAAATCAGGCAAAGTGAGACAT	100 bp	NlaIII	C	T	C
**PCR conditions**	94°C 3′; 94°C 30″, 55°C 30″, 72°C 30″, ×25 cycles; 72°C 5′; 4°C for storage.					
**M7**	F:TGTACCCTTGACCAATGCCTTR:TTGTAGTTGAGTTACTGTTCTTCTA	126 bp	BfaI	C	G	C
**M88**	F:CTGTAGCCTAGAGCC TGCCAAAR:TAGAGAGGAAAACCTATCTTGGATG	161 bp	HhaI	A	G	G
**M95**	F:ATAAGGAAAGAC TACCATATTAGC GR:TTTGAAGGCCCCAGTTGTGAG	202 bp	HhaI	C	T	C
**M119**	F:AGGTAAATGACTCACCCTAAGGAAGR:GGGTTATTCCAATTCAGC ATACACGC	161 bp	BstuI	A	C	C
**M120**	F:TGGACAGATTACAGTAAACCTTCAACR:GTATAATTTCCCTTAAAAACATCATG	123 bp	BspHI	T	C	T
**M122**	F:TAGAAA AGCAATTGAGATACTAATTCAR:GCGATGCTGATATGCTAGTTCAG	122 bp	NlaIII	C	T	C
**PCR conditions**	94°C 3′; 94°C 30″, 55°C* 40″, 72°C 40″, ×25 cycles; 72°C 5′; 4°C for storage.					

**Table 7:** Information for six Y-SNP and seven Y-STR fluorescence primers.

Loci	Primer sequence (5′→3′)	Fluorescence	Product length (bp)	
			Wild type	Mutant type
**M175**	F: TTGAGCAAGAAAAATAGTACCCAR: TTCAGTTAGCCTTGATTGACTGT	FAM	226	221
**M121**	F: ACAAAGACCTGGACAGATTAC R: CCCTTAAAAACAGCATGATA	FAM	123	118
**M134**	F: AGAATCATCAAACCCAGAAGGR: TCTTTGGCTTCTCTTTGAACAG	NED	232	231
**M117**	F: TACGAAGAAAATCAAGGCTATTAR: TTGGGTAGAAAAACTGCAAGTAG	FAM	317	313
**M111**	F: TAACATAAACAGTATGCCAAAR: TGCCCTAAAGTTAATACCAG	NEX	197	195
**M15**	F: ACAAATCCTGAACAATCGC-R: GTCTGGGAAGAGTAGAGAAAAG-	FAM	151	142
**PCR conditions** #	94°C 3′; 94°C 30″, 61°C * 40″, 65°C 1′; ×13 cycles (*,−0.5°C/cycle); 94°C 30″, 56°C * 40″, 65°C 1′, ×20 cycles; 65°C 5′; 4°C for storage.			
**DYS388**	F:GTGAGTTAGCCGTTTAGCGAR:CAGATCGCAACCACTGCG	FAM		
**DYS389**	F:CCAACTCTCATCTGTATTATCTATGR:TCTTATCTCCACCCACCAGA	FAM		
**DYS390**	F:TATATTTTACACATTTTTGGGCCR:TGACAGTAAAATGAACACATTGC	NED		
**DYS391**	F:CTATTCATTCAATCATACACCCAR:GATTCTTTGTGGTGGGTCTG	HEX		
**DYS392**	F:TCATTAATCTAGCTTTTAAAAACAAR:AGACCCAGTTGATGCAATGT	NED		
**DYS393**	F:GTGGTCTTCTACTTGTGTCAATACR:AACTCAAGTCCAAAAAATGAGG	HEX		
**DYS394**	F:CTACTGAGTTTCTGTTATAGTR:ATGGCATGTAGTGAGGACA	HEX		
**PCR conditions** #	94°C 1′; 94°C 20″, 61°C * 40″, 65°C 30″; ×14 cycles (*,−0.5°C/cycles); 94°C 20″, 54°C * 40″, 65°C 30″, ×25 cycles; 65°C 5′; 4°C for ever.			

# touch-down PCR.

### Data analysis

The Y-SNP haplogroup of every individual was defined according to the genotyping results and Fig. 7. Y-chromosome haplogroups can be considered as a monophyletic clade in the phylogenetic tree (i.e., a set of haplogroups comprising all descendants of their most recent common ancestor, inferred from the shared mutations). For example, O3*, O3e*, and O3e1* all share a T→C mutation at locus M122. O3* is the ancestral haplogroup of the M122C alleles, whereas O3e* and O3e1* are the 2 derived haplogroups with additional mutations, M134 and M117, respectively. Haplogroup frequencies were calculated and compared among the three EC high-risk populations. Chi-square tests were performed to evaluate the differences in haplogroup frequency among populations.

PC analysis and correlation analysis were performed to compare the genetic affinity among the three EC high-risk populations and other groups. The study populations were defined as northern East Asian (NEAPs) and southern East Asian populations (SEAPs) according to their geographic locations. The Yangtze River was used as the geographic border to separate the NEAPs and SEAPs; NEAPs were further divided into northern Hans (NHs) and northern minority nationalities (NMNs), and SEAPs were defined as southern Hans (SHs) and southern minority nationalities (SMNs). Additional Y-SNP data allowed for dividing the major Chinese Han populations into 2 groups (for detailed comparative data, see Table 5): NHs, including Hebei [17], Liaoning, data provided by the State Key Laboratory of Genetic Engineering and Center for Anthropological Studies (School of Life Sciences, Fudan University), Xinjiang, Gansu, Shanxi, Neimeng [16], Shandong and Henan [17] populations; and SHs, including Hakka [18], Hunan, Hubei, Zhejiang, Jiangxi, Shanghai, Anhui, Jiangsu, Yunnan, Sichuan [17], Guangzhou and Guangxi populations, data provided by the State Key Laboratory of Genetic Engineering and Center for Anthropological Studies (School of Life Sciences, Fudan University). Added to the analysis were Y-STR data for 16 populations, which were the total populations that we could obtain from references. Y-STR data allowed for dividing these populations into 4 groups: SHs (Fujian [35], Anhui [36], Yunnan [37], Zhejiang [20] and Guangzhou [38] Han (belong to Guangfu clans), labeled 4−6, 8−9); NHs (Henan [21], Dongbei [22], Beijing [39] and Tianjing [23] Han; NMNs (Tibetan [25], Uygur, Krigiz, Manchu[40], and Shui [41]; and SMNs (Naxi [42] and, Zhuang [40]. The EC high-risk CSP, FJP, and HTMP belong to SHs, SHs, and NHs, respectively. The Guangzhou Han, Hakka, and the EC high-risk CSP belong to the Guangfu, Hakka, and Fulao clans, respectively, which are the three major clans in Guangdong Province. They are geographically proximate, so the Guangzhou Han (labeled 15 in Fig. 2 and 9 in Fig. 3) and Hakka populations (labeled 12 in Fig. 2) were also included in the PC analysis.

The paternal genetic relationship among the EC high-risk populations was further characterized by hierarchical cluster analysis. The extent of genetic differentiation of the populations was estimated by the Rst statistic on the basis of the Y-STR haplotypes. A neighbor-joining tree was constructed according to the Rst distance matrix to show the phylogenetic structure among the populations.

STRs can infer minute genetic diversity within a haplogroup in different populations, so the network was constructed using Network 4.516 software (www.fluxus-engineering.com) with Y-STR data from individuals of the EC high risk CSP, FJP and HTMP based on Y-SNP information. In the network map, individuals with the same mutations of Y-STRs were present in the same node and one node can generate other nodes below due to gradual Y-STR mutations.

## References

[1] Su B, Xiao JH, Underhill P, Deka R, Zhang WL (1999). Y-chromosome evidence for a northward migration of modern humans in Eastern Asia during the last ice age.. Am J Hum Genet.

[2] Parkin DM, Bray F, Ferlay J, Pisani P (2001). Estimating the world cancer burden: Globocan 2000.. Int J Cancer.

[3] Stewart BW, Kleihues P (2003). World cancer report..

[4] Roohullah, Khursheed MA, Muhanmmad AS, Zainullah K, Haider SW (2005). An alarming occurrence of Esophageal cancer in Balochistan.. Pakistan J Med Res.

[5] Su M, Liu M, Tian DP, Li XY, Yang HL (2007). Temporal trends of esophageal cancer during 1995-2004 in Nanao Island, an extremely high-risk area in China.. Eur J Epidemiol.

[6] Chen WS, Yang HL, Cai WS, Qin JW, Zhang C (1996). Epidemiologic features of esophagus cancer in Nanao county Guangdong province from 1987 to 1992.. Chin J Cancer.

[7] Peng XE, Yang XH, Shi XS, Ma HB, Hu QB (2003). Study on geographic characteristics of esophageal cancer in Fujian province.. Chin J Prev Contr Chron Non-commun Dis.

[8] Zou XN, Lu FZ, Zhang SW (2002). Characteristics of esophageal cancer mortality in China, 1990-1992.. Bull Chin Cancer.

[9] Su M, Li XY, Tian DP, Wu MY, Wu XY (2004). Clinicopathologic analysis of esophageal and cardiac cancers and survey of molecular expression on tissue microarrays in Chaoshan littoral of China.. World J Gastroenterol.

[10] Huang ZF (2000). Ancestor of Chaoshan..

[11] Huang T (1999). Origin and development of Chanshan culture..

[12] Su M, Lu M, Li XY, Zhang GH, Yang HL (2005). A genetic epidemiological study on esophageal cancer of Nanao island in Chao-shan region [A]. Proceedings of the first assembly international academic conference of esophagus carcinoma [C]..

[13] Hu N, Dawsey SM, Wu M, Bonney GE, He LJ (1992). Familial aggregation of oesophageal cancer in Yangcheng County, Shanxi Province, China.. Int J Epidemiol.

[14] Chang-Claude J, Becher H, Blettner M, Qiu S, Yang G (1997). Familial aggregation of oesophageal cancer in a high incidence area in China.. Int J Epidemiol.

[15] Jin L, Su B (2000). Natives or immigrants: modern human origin in East Asia.. Nat Rev Genet.

[16] Wen B (2004). Y-chromosome and mtDNA polymorphism and genetic structure of East Asian..

[17] Ke YH, Su B, Xiao JH, Chen H, Huang W (2001). Y-chromosome haplotype distribution in Han Chinese populations and modern human origin in East Asians.. Sci in China (Series C).

[18] Li H, Pan WY, Wen B, Yang NN, Jin JZ (2003). Origin of Hakka and Kakkanese: a genetic analysis.. Acta Genetica Sinica.

[19] Yu JK, Sun H, Shi L, Shi L, Qian YP (2006). A polymorphism analysis of 17 biallelic marker on Y chromosome in 5 Chinese populations.. Chin J Appl Environ Biol.

[20] Wu WW, Zheng XT, Pan LP, Hao HL, Fu T (2005). A study of polymorphisms of 16 Y-STR loci in Han population in Zhejiang.. Forensic Sci Technol.

[21] Feng CJ, Xiang ZD, Shen CB (2005). Polymorphisms of twelve Y-chromosome STR loci in Han population in Henan.. Forensic Sci Technol.

[22] Ba GJ, Lin ZQ, Li S (2007). Polymorphisms of eleven Y-chromosome STR loci in Han population in Northeast of China.. J Forensic Med.

[23] Kuang JZ, Zhu W, Nie TG, Liu Y, Liu MN (2005). Polymorphisms of 12 Y-STR loci in Han population in Tianjin.. Forensic Sci Technol.

[24] Chen SQ, Chen HJ, Zeng XG, Li Q, Zhu ZL (2005). Polymorphism analysis of seven Y-STR loci in Han population in Hunan.. Chin J Forensic Med.

[25] Zhao JM, Yuan DY, Kang LL, Liu K, Li SB (2007). Genetic polymorphisms of 14 Y-chromosomal short tandem repeat loci and haplotypes in Tibetan.. Chin J Med Genet.

[26] Si MQ (2007). History as a Mirror.. Chinese Publishing House.

[27] Xie CG (2003). The transplantation and spreading of Min-nan Culture of Song dynasty in Chao-shan areas.. J Hanshan Teachers College (China).

[28] Chen ZH (2001). Outline of Chaoshan culture..

[29] Fei XT (1999). The Pattern of Diversity in Unity of the Chinese Nation..

[30] Ge JX, Wu SD, Cao SJ (1997). The migration history of China..

[31] Sambrook J, Fritsch EF, Maniantis T (Translated by Jin Dong-Yan, Li Meng-feng) (1996). Molecular Cloning: A Laboratory Manual. 2 ^nd^ ed..

[32] Underhill PA, Jin L, Lin AA, Mehdi SQ, Jenkins T (1997). Detection of numerous Y chromosome biallelic polymorphisms by denaturing high-performance liquid chromatography.. Genome Research.

[33] Wen B, Shi H, Ren L, Xi HF, Li KY (2003). The origin of Mosuo people as revealed by mtDNA and Y chromosome variation.. Sci in China (Series C).

[34] The Y chromosome Consortium (2002). A nomenclature system for the tree of human Y-chromosomal binary haplogroups.. Genome Res.

[35] Li B, Hu SP, Liu JW (2006). Polymorphisms of 12 Y-STR loci in Han population in Minnan area.. Chin J Forensic Med.

[36] Wang DH, Chen L, Hou QT, Yang YL (2006). Polymorphisms of 11 Y-STR loci in Han population in Anhui.. Chin J Forensic Med.

[37] Zhang XH, Wu WW, Tang JX, Qian GL, Zhang XM (2006). Polymorphisms of Eleven Y- chromosome STR loci and For ensic Application in Yunnan Han Population.. J Forensic Med.

[38] Liu C, Chen L, Chen XH, Liu CH, Wang HJ (2007). Polymorphism of 12 Y-STR loci in Guangzhou Han population and its application in forensic medicine.. J South Med Univ.

[39] Yang J, Liu YC, Tang H, Huo ZY (2002). Polymorphisms of Y-chromosome STR loci in Beijing population.. Chin J Forensic Med.

[40] Yu L, Huang XQ, Shi L, Shi L, Yu JK (2005). Gene frequencies and haplotypes of nine Y-short tandem repeat loci in four minority populations of China.. Chin J Med Genet.

[41] He Y, Shan KR, Xie Y, Xiu J, Wu CX (2006). Polymorphism analysis of seven Y-STR loci in Shui population in Guizhou, China.. Hereditas (Beijing).

[42] Yang XX, Yang ZL, Shi H, Gao L, Dong YL (2005). Study of Y-STR genetic polymorphism for Naxi nationality.. J Dali University.

